# Estimating Fiber Orientation Distribution Functions in 3D-Polarized Light Imaging

**DOI:** 10.3389/fnana.2016.00040

**Published:** 2016-04-19

**Authors:** Markus Axer, Sven Strohmer, David Gräßel, Oliver Bücker, Melanie Dohmen, Julia Reckfort, Karl Zilles, Katrin Amunts

**Affiliations:** ^1^Research Centre Jülich, Institute of Neuroscience and MedicineJülich, Germany; ^2^Jülich Supercomputing Centre, Institute for Advanced Simulation, Research Centre JülichJülich, Germany; ^3^Research Centre Jülich, Simulation Lab Neuroscience, Bernstein Facility for Simulation and Database Technology, Institute for Advanced SimulationJülich, Germany; ^4^Department of Psychiatry, Psychotherapy, and Psychosomatics, RWTH Aachen UniversityAachen, Germany; ^5^JARA Jülich-Aachen Research Alliance, Translational Brain MedicineAachen, Germany; ^6^C. and O. Vogt Institute for Brain Research, Heinrich-Heine-University DüsseldorfDüsseldorf, Germany

**Keywords:** connectome, fiber architecture, human brain, polarized light imaging, 3D-PLI, ODF

## Abstract

Research of the human brain connectome requires multiscale approaches derived from independent imaging methods ideally applied to the same object. Hence, comprehensible strategies for data integration across modalities and across scales are essential. We have successfully established a concept to bridge the spatial scales from microscopic fiber orientation measurements based on 3D-Polarized Light Imaging (3D-PLI) to meso- or macroscopic dimensions. By creating orientation distribution functions (pliODFs) from high-resolution vector data via series expansion with spherical harmonics utilizing high performance computing and supercomputing technologies, data fusion with Diffusion Magnetic Resonance Imaging has become feasible, even for a large-scale dataset such as the human brain. Validation of our approach was done effectively by means of two types of datasets that were transferred from fiber orientation maps into pliODFs: simulated 3D-PLI data showing artificial, but clearly defined fiber patterns and real 3D-PLI data derived from sections through the human brain and the brain of a hooded seal.

## Introduction

The repertoire of neuroimaging tools that are able to target neuronal connectivity in both the living and the post mortem brain, is continuously growing. Technological developments in particular in the field of microscopy (Osten and Margrie, 2013), new preparation and labeling methods (Chung et al., 2013; Costantini et al., 2015) and a better understanding of how to process the collected data (Amunts et al., 2013; Silvestri et al., 2015), facilitates this advancement (for recent review Amunts and Zilles, 2015). Several of these techniques address either cellular or even molecular dimensions, e.g., light sheet microscopy, or they provide data at meso- to macroscopic scales, e.g., Diffusion Magnetic Resonance Imaging (dMRI). Thus, the data output generated by different technical approaches and imaging techniques results in different data types, formats, and sizes, and is obtained on different spatial scales. As a consequence, comprehensible comparison across modalities and across scales evolves into a basic necessity for the neuroscience community.

In the present study, we demonstrate how to bridge the spatial scales from microscopic post mortem fiber visualization and orientation measurements based on 3D-Polarized Light Imaging (3D-PLI; Axer et al., 2011a,b) to meso- or macroscopic dimensions as targeted by dMRI. Our approach enables the propagation of the entire information on the microscopic fiber architecture within individual voxels by means of sophisticated data fusion. Based on the commonly used strategy to integrate vector data into a comprehensive description by employing Orientation Distribution Functions (ODFs, Bunge, 1982), we introduce the *pliODF* derived from 3D-PLI. pliODFs benefit from the unique property of 3D-PLI to extract high-resolution 3D vector fields indicating the spatial orientation of single fibers and fiber tracts in unstained brain sections. By transferring the entire vector data within a certain compartment, the so-called *super-voxel*, into a 3D statistical description (often visualized in form of a glyph), an efficient downscaling of high-resolution vector-like data becomes feasible. Considering the pure size of a 3D-PLI dataset that covers a whole human brain (i.e., 2500 sections scanned at 1.3 microns pixel size sum up to at least 500 TByte), the development of a method that reliably resamples large-scale microscopic data is of particular importance.

The choice of using ODF-like statistical descriptions of multiple-fiber compartments is based on the fact, that recent dMRI methods such as Diffusion Spectrum Imaging, Q-ball Imaging or Spherical Deconvolution (Tuch et al., 2002; Alexander, 2005; Wedeen et al., 2005; Dell'Acqua et al., 2013) also approximate the distribution of fiber orientations within an MRI-voxel by means of fiber Orientation Distribution Functions (fODF; Alexander et al., 2002; Hess et al., 2006; Rathi et al., 2009; Assemlal et al., 2011). Using a similar mathematical description is clearly beneficial for multimodal comparisons. Approaches have been reported aiming at the extraction of textural 2D-information, i.e., local fiber orientations, from images of histological brain sections stained for myelin in order to create 2D structural ODFs (Leergaard et al., 2010; Budde and Frank, 2012). In this context, small regions of interest were successfully compared to fODFs obtained from dMRI measurements. These studies represent important steps toward a region-based comparison of 2D fiber architecture obtained from different modalities. However, the 3D fiber architecture across large datasets has not been addressed yet.

Here, we benefit from the three-dimensional nature of the polarized light imaging approach, which provides measurements of the 3D fiber structures at the level of individual brain sections. Consequently, a super-voxel—and also a pliODF—can arbitrarily be composed of compartments within a section, but also across aligned neighboring sections without the requirement to change the software tools. To demonstrate the validity of our approach, two types of 3D-PLI datasets were transformed into pliODFs: (i) simulated 3D-PLI data showing synthetic, but clearly defined fiber-like patterns and (ii) real 3D-PLI data derived from sections through the human brain and the brain of a hooded seal. The human brain data were selected to highlight the gain of high-resolution imaging of brain regions with challenging fiber compositions such as the complex fiber crossings in the corona radiata or low fiber density regions in the cortex. The chiasm of the hooded seal with its nearly perpendicularly decussating fiber tracts (cf. Dohmen et al., 2015) appeared to be well suited to show the transition from simulation-based ODF generation to the most simple crossing fiber tract constellation observable in real brain tissue.

## Materials and methods

**Figure 1:**
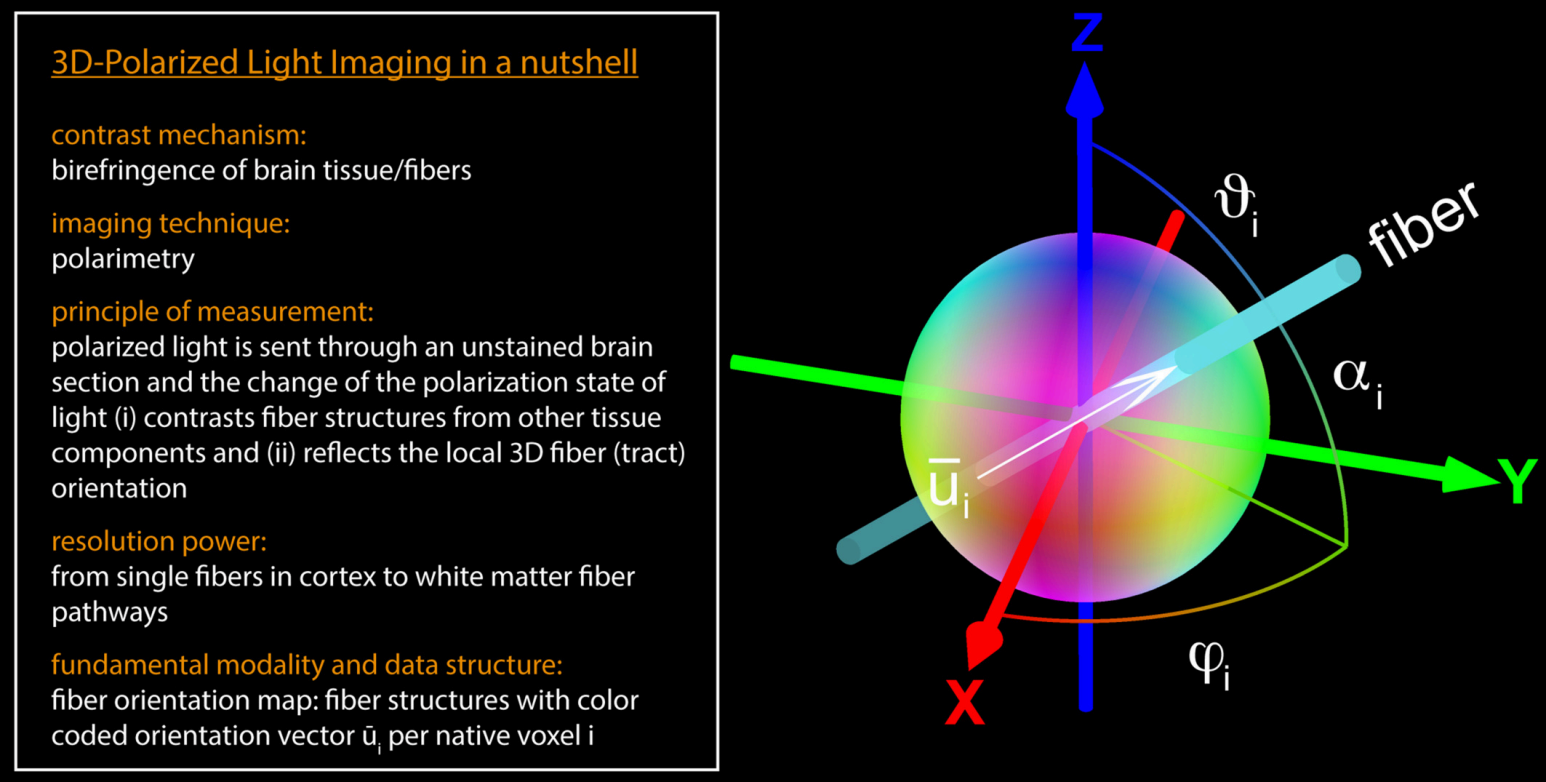
**3D-PLI in a nutshell**. The fiber orientation vector ${\vec{u}}_{i}$ located at the native voxel location *i* is defined by the direction angle φ_*i*_ and the inclination angle α_*i*_. The brain section is aligned to the x-y-plane (at *z* = 0). In general, a unit vector can be described as a distinct point on a unit sphere. For 3D-PLI, the determined unit vector represents an axis in space (in the mathematical and the anatomical sense), since the initial and the terminal points cannot be assigned uniquely. Therefore, the point reflection across the center of the sphere has to be assigned to the same vector. For visualization purposes, the vectors were encoded in RGB color space: the x-component of ${\vec{u}}_{i}$ is represented by the red color channel (R), the y-component by the green color channel (G), and the z-component by the blue color channel (B).

3D-PLI (Axer et al., 2011a,b; Zeineh et al., 2016) has demonstrated its unique capabilities (i) to reveal fiber structures at multiple scales, such as long-range connections and even single fibers and crossings within unstained histological brain sections, and (ii) to determine spatial fiber orientations (i.e., 3D unit vectors down to the scales of fiber diameters (0.4–15 μm). 3D-PLI is applicable to unstained microtome sections of post mortem brains and utilizes the optical birefringence of nerve fibers, which basically arises from the highly ordered arrangement of lipid molecules in the myelin sheath surrounding most of the axons in the brain. Polarimetric setups (e.g., a polarizing microscope) are employed to carry out birefringence measurements and to give contrast to individual nerve fibers and their tracts. Supported by fundamental principles of optics and dedicated simulation approaches (Dohmen et al., 2015; Menzel et al., 2015), the measured signals are additionally interpreted in terms of spatial fiber orientations by means of unit orientation vector descriptions (Figure 1). The algorithms used for the fiber orientation interpretation have been implemented as an automated 3D-PLI analysis workflow suitable for distributed supercomputing, as described by Axer et al. (2011b); Amunts et al. (2014).

### The fiber orientation map

Application of the 3D-PLI analysis workflow results in a unit vector-based description of the fiber orientation determined for each tissue voxel, referred to as a native voxel. The native voxel dimensions are defined by the image pixel size provided by the optical setup and the thickness of the studied histological brain section (70 μm in the present study). The generation of pliODFs was exclusively performed on 3D-PLI datasets with native voxel dimensions of 1.3 × 1.3 × 70 μm. Each orientation vector reflects the net effect of all fibers comprised within a voxel. The assembly of all unit vectors represents the fiber orientation map (*FOM*).

The orientation unit vector ${\overset{⇀}{u}}_{i}$ at voxel location *i* can be parameterized by two angles, i.e., by spherical coordinates: the direction angle φ_*i*_, which represents the projection of the principal fiber axis within the sectioning plane, and the inclination angle α_*i*_, which is the angle between the principal fiber axis and the sectioning plane (Figure 1 and Equation 1).

(1) $$\begin{array}{l} {\overset{⇀}{u}}_{i}=\left(\begin{array}{c} cos\alpha_{i}\cdot cos\varphi_{i}\\ cos\alpha_{i}\cdot sin\varphi_{i}\\ sin\alpha_{i} \end{array}\right) \end{array}$$

### Image generation and data acquisition

#### Simulated 3D-PLI data

The simulation software tool SimPLI (Dohmen et al., 2015; Menzel et al., 2015) was used to generate two synthetic 3D-PLI datasets with known configurations of fiber-like structures. In SimPLI, three main steps of simulation are implemented: (i) the generation of an arbitrary spatial arrangement of synthetic fibers and the discretization into a three-dimensional fiber orientation vector field with a certain resolution (e.g., 70 μm isotropic), (ii) the calculation of the transmitted light intensity based on the Jones matrix calculus (Jones, 1941) yielding a synthetic 3D-PLI image series, and (iii) the simulation of environmental clutter arising from the camera and the tissue in the optical path by adding blurring and noise effects.

**Figure 2:**
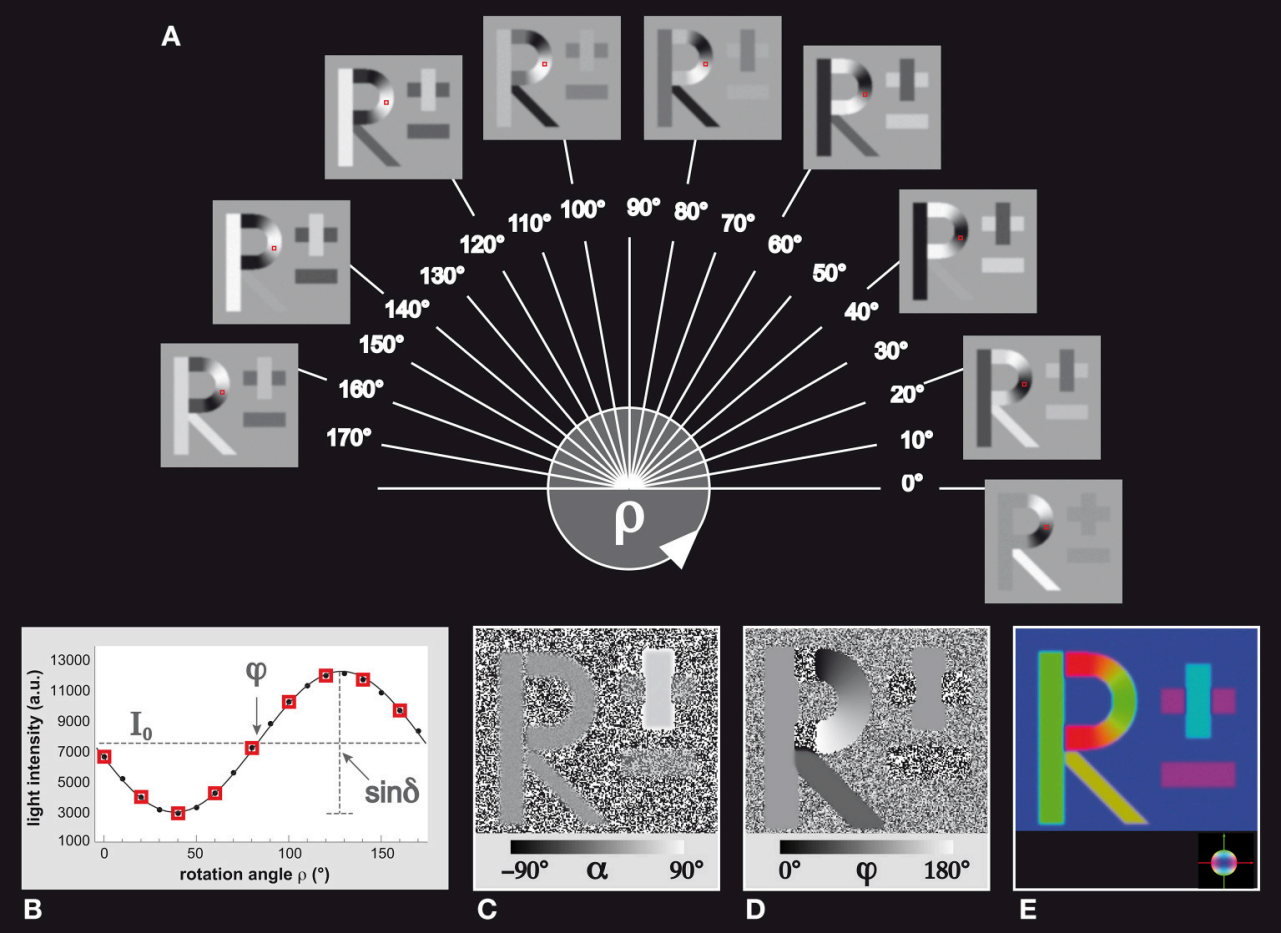
**Simulated 3D-PLI reference dataset. (A)** The standard 3D-PLI measurement yields 18 images corresponding to equidistant rotation angles ρ between 0° and 170°. Here, a selection of nine generated images of simulated birefringent structures (the letter *R* and the ± sign) is shown. Each image has a size of 200 × 200 pixels. The varying pixel intensities are comparable to observed signals in measurements of brain sections. The red squares indicate a native voxel of interest that is displayed **(B)** in terms of the observed light intensity as a function of the rotation angle. The physical model that underlies 3D-PLI provides a sinusoidal description of the simulation (continuous black line), and relates **(C)** its amplitude to the inclination angle α via the retardation value *sinδ*and **(D)** its phase to the direction angle φ. The introduced effects of blurring and noise are evident: the minus sign in the direction map **(D)**, for example, shows direction angles that are spread around the initial direction φ = 0° by±2.5°. In a π-periodic system, this is equivalent to an angle range between 177.5° and 2.5°. **(E)** Visualization of the FOM with the determined vectors $\vec{\text{u}}$ encoded in RGB color space (see color sphere for the relation between orientation and color-coding).

**Figure 3:**
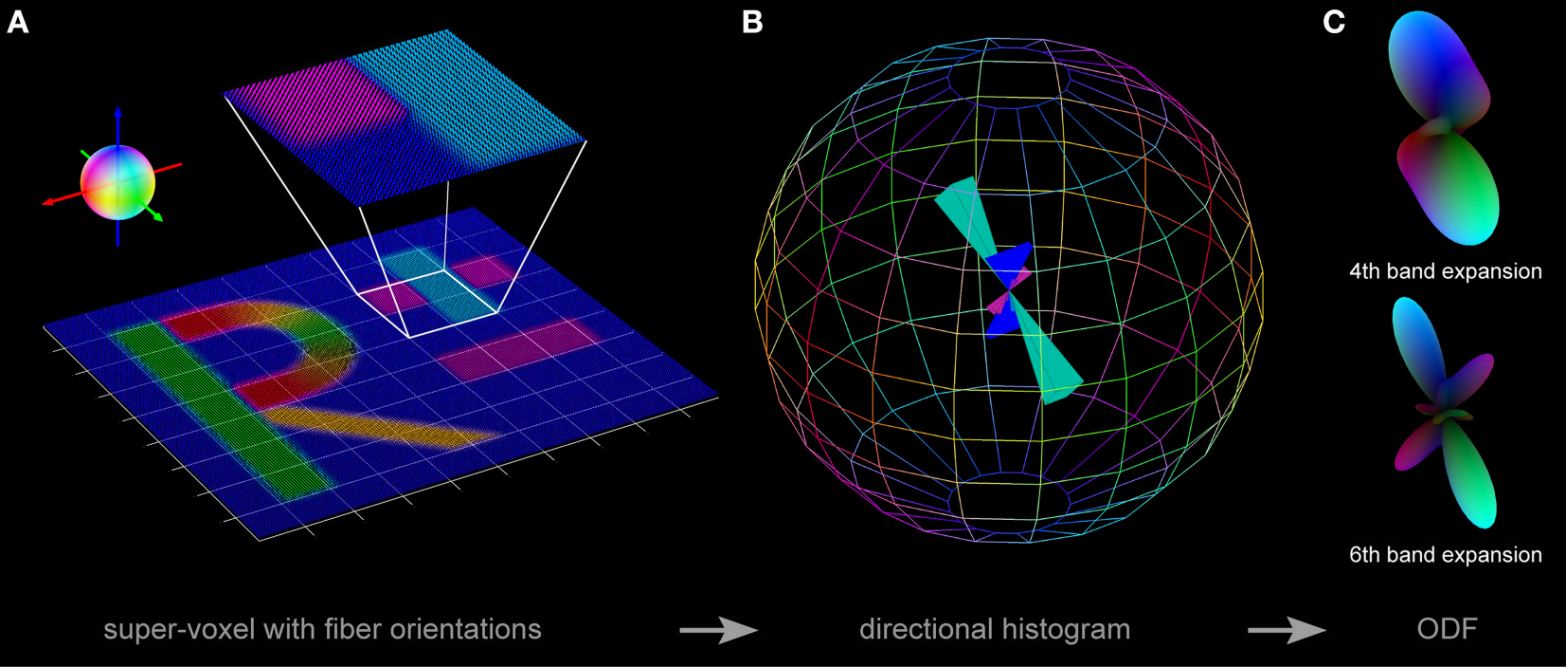
**Three steps toward pliODF generation. (A)** First, a FOM is divided into regular domains or super-voxels. The exemplarily enlarged super-voxel contains 40 × 40 × 1 native voxels representing three predominant fiber orientations, which show a relative frequency of occurrence of ~¼(blue color), ~¼(magenta color) and ~½(cyan color), respectively. The color sphere defines the relation between orientation and color-coding. **(B)** Second, a normalized directional histogram with a discretized binning on a unit sphere is created for each super-voxel. The relative fraction of fiber orientations assigned to a particular bin is reflected by the length of the colored solid angle originating from the middle of the sphere. The symmetry of the histogram with respect to point reflection across the center of the sphere is evident. Here, the total number of bins distributed over the sphere was set to 164 and the three predominant input fiber orientations are still preserved. **(C)** Third, a spherical harmonics expansion is used to approximate each directional histogram. Depending on the selected depth of expansion (e.g., to the 4th or the 6th band), orientation distribution features might become occluded by interpolation.

In order to validate the different methodological steps employed to transfer a FOM into a set of orientation distribution functions, a well-defined template providing unambiguous structural macroscopic and microscopic features in terms of left/right, top/down and in-plane/out-of-plane orientations, was required. This dataset generated by means of SimPLI is shown in Figures 2A–D. It is composed of a stack of 18 images and comprises birefringent “fibers” forming human readable structures (“fiber bundles”), such as the capital letter “*R”* and the “±” sign. The line thickness of the letters (or the thickness of the “fiber bundles”) was chosen to be 20 pixels on average. The fiber inclination angles in “*R”* were all set to α = 0°, while the inclination angles were set to α = +45° and α = −45° for the “+” and “−” sign, respectively. The direction angles φ were aligned with the local structures using a right-handed coordinate system, i.e., the horizontal components (e.g., the “−“ sign) are identified by φ = 0° while the vertical components are represented by φ = 90°. The diagonal element of the “*R”* has a direction of φ = 135°. The background is composed of 90° inclined fibers corresponding to light intensity variations equal to zero. This dataset was subjected to the 3D-PLI analysis workflow to extract the corresponding FOM (Figures 2E, 3A).

A second, more realistic dataset was generated with SimPLI and subjected to 3D-PLI analysis (cf. **Figure 5F**), and has already been described in detail in Dohmen et al. (2015): the model of a chiasm of the hooded seal. This data bridges to the successive section on real 3D-PLI data sets.

#### 3D-PLI data obtained from histological sections of the hooded seal and the human brain

Two human brains and the optic chiasm of a hooded seal were immersed in 4% paraformaldehyde. After cryoprotection (with a 20% glycerin solution), the brain tissue was deep frozen at −70°C and stored at the same temperature till further processing. A whole human brain and the occipital lobe of a human brain were coronally sectioned and optic chiasm was axially sectioned using a large-scale cryostat microtome (Polycut CM 3500, Leica, Germany), and eventually coverslipped with a 20% glycerin solution. The chosen section thickness was 70 μm. During sectioning, each blockface was imaged using a CCD camera mounted above the brain in order to obtain an undistorted reference for each section (cf. **Figures 5A**, **6A**, **7A**). No staining was applied. In each case, this procedure resulted in a complete series of sections through large tissue samples, which enables a 3D reconstruction. The brains were acquired in accordance with local legal and ethical requirements.

The brain sections were measured in two custom-made polarimetric setups, the large-area polarimeter and the polarizing microscope, providing images with pixel dimensions of 64 × 64 μm and 1.3 × 1.3 μm, respectively (for technical details refer to Axer et al., 2011b). The datasets were passed to the 3D-PLI analysis workflow to extract the corresponding FOMs. For pliODF generation only the high-resolution data were used, while the 64 μm-sized FOMs were used for plausibility checks and reference measures.

#### Estimation of fiber orientation distribution functions (pliODFs)

The fundamental aim was to generate a mathematical description -the orientation distribution function (pliODF)– that quantifies the spatial distribution of fiber orientations determined by 3D-PLI within a rectangular compartment. The compartment can be defined in a single FOM or in a series of FOMs and is referred to as super-voxel. A super-voxel is composed of (*r* × *c* × *s* = *rows* × *columns* × *sections*) native voxels. In order to calculate pliODFs, fundamental approaches from material science (texture analysis in crystallography; Bunge, 1982) and directional statistics (Mardia and Jupp, 2000) were applied and adopted to the needs of 3D-PLI.

The implemented procedure was based on three steps (cf. Figure 3):

- the definition of super-voxels (rectangular compartments) in high-resolution FOMs to resample the dataset,
- the collection of all fiber orientation vectors comprised in a super-voxel to derive the discretized distribution of orientations by creation of a normalized directional histogram on a unit sphere, and
- the approximation of the orientation probability distribution density by fitting the directional histogram with a series expansion using spherical harmonics.

In the following, steps (A) to (C) will be explained in more detail. As mentioned above (Figure 1), a 3D-PLI orientation vector can be expressed in spherical coordinates (the polar angle $\vartheta =\frac{\pi }{2}-\alpha$ and the azimuth angle φ) and defines two points on the unit sphere S^2^. This feature was used to construct a directional histogram aiming at a statistical description of the fiber orientations contained in a super-voxel. To discretize the distribution of fiber orientations, the surface of a unit sphere was subdivided into planar bins (bin centers characterized by latitude and longitude) with a total bin number *n* calculated by

(2) $$\begin{array}{l} n=\left(\#\;of\;latitudes\right)+\left(\#\;of\;longitudes\right)+2\;polar\;caps. \end{array}$$

*n* was adopted to the specific requirements of the used datasets. In a final step, the number of vectors falling into each bin was determined (Figure 3B).

Normalization of the directional histogram enabled to mathematically describe the empirical orientation probability distribution density *p(ω)* by

(3) $$\begin{array}{l} \frac{dN}{Z}=p\left(\omega \right)d\Omega , \end{array}$$

with *Z* being a normalization factor ($Int\left(\frac{dN}{Z}\right)=1$), dΩ the dihedral angle differential, and ω the spatial direction. *p(ω)* was expanded into a series of generalized spherical harmonics:

(4) $$\begin{array}{r} p\left(\omega \right)=\overset{\infty }{\underset{l=0}{\sum }}\overset{l}{\underset{m=-l}{\sum }}\overset{l}{\underset{n=-l}{\sum }}C_{l}^{mn}T_{l}^{mn}\left(\omega \right)\\ =\overset{\infty }{\underset{l=0}{\sum }}\overset{l}{\underset{m=-l}{\sum }}\overset{l}{\underset{n=-l}{\sum }}C_{l}^{mn}{e^{I\varphi_{2}}P}_{l}^{mn}\left(\Phi \right)e^{I\varphi_{1}}. \end{array}$$

The $P_{l}^{m}$ represent the associated Legendre polynomials. Due to the existing rotational symmetry (i.e., the determined fiber orientations are invariant with respect to rotation around the axis defined by the unit vector), the general description of the series simplified to an expansion in terms of spherical harmonics $Y_{l}^{m}\left(\vartheta ,\varphi \right)$:

(5) $$\begin{array}{l} p\left(\vartheta ,\varphi \right)=\overset{\infty }{\underset{l=0}{\sum }}\overset{l}{\underset{m=-l}{\sum }}C_{l}^{m}Y_{l}^{m}\left(\vartheta ,\varphi \right), \end{array}$$

with *l* and *m* denoting the band index and sub-band index, respectively. A series expansion to the 6th band, for example, means *l* = 0, …, 6 and −*l* ≤ *m* ≤ l (with 1 + 3 + 5 + 7 + 9 + 11 + 13 = 49 members).

The expansion of the empirical orientation probability distribution *p*(ϑ, φ) into real valued symmetric spherical harmonics series reads as:

(6) $$\begin{array}{l} p\left(\vartheta ,\varphi \right)=\overset{\infty }{\underset{l=0}{\sum }}\overset{2l}{\underset{m=-2l}{\sum }}C_{2l}^{m}y_{2l}^{m}\left(\vartheta ,\varphi \right)\approx \overset{\hat{L}}{\underset{l=0}{\sum }}\overset{2l}{\underset{m=-2l}{\sum }}C_{2l}^{m}y_{2l}^{m}\left(\vartheta ,\varphi \right), \end{array}$$

with

(7) $$\begin{array}{l} \frac{1}{2}\left(L+1\right)\left(L+2\right)=\left(2\hat{L}+1\right)\left(\hat{L}+1\right) \end{array}$$

coefficients (with $2\hat{L}=L$).

To generate a pliODF, the coefficients $C_{2l}^{m}$ of the expansion up to the *l*^th^ band had to be determined. This was realized using a least square fit algorithm, because of its numerical stability and the efficiency of the numerical implementation. Due to discretization and truncated series expansion, the pliODF only approximates the empirical orientation probability distribution *p*.

#### Computing

Both, the size of the processed data and the computationally intensive algorithms to determine the expansion coefficients required a supercomputing environment. For this reason, we used the Juelich Dedicated GPU Environment (JuDGE), hosted by the Jülich Supercomputing Center (JSC), Germany. It was equipped with 206 compute nodes, where each node consisted of two Intel Xeon Westmere 6-core processors operating at 2.66 GHz. Each compute node contained 96 GB of main memory. Per node there were either two NVIDIA Tesla M2050 (Fermi) GPUs or two NVIDIA Tesla M2070 (Fermi) GPUs integrated. The inter- and intra-node communication was realized by the message passing interface (MPI) protocol.

Runtime measurements were performed for a region of interest in a FOM gained from the human brain tissue [i.e., ROI (1) as depicted in **Figure 6B**], which was composed of 3712 × 4576 orientation vectors. The number of compute cores was set to 72 in order to keep the compute time in an acceptable range, but to highlight the differences in run-time properly at the same time. The number of bands and the size of super-voxels were taken as parameters.

#### Visualization

ODFs are typically visualized either (i) by means of a textured sphere, where the color of a point on the surface represents the probability of its corresponding orientation, or (ii) by simply scaling the surface with the probability *p*. To visualize the generated pliODFs, the second option was applied in combination with the color-coding scheme also used to visualize different fiber orientations in a FOM and the surface intensity increasing with the probability (Figure 3C). The scheme is based on the RGB color space with the red channel representing the x-component, the green channel representing the y-component, and the blue channel representing the z-component of the fiber orientation in the reference coordinate frame. The peaks of the pliODF shape and the color-coding reflect the most common fiber directions such that both the pliODFs and the FOMs are visually comparable.

### Results

#### Simulated 3D-PLI data

The FOM obtained from the simulated dataset was divided into super-voxels of 10 × 10 × 1, 20 × 20 × 1, and 40 × 40 × 1 native voxels, respectively, to create pliODFs. These clusters were chosen (i) to demonstrate the accurate interpretation of the input vectors including the conservation of the coordinate system and (ii) to investigate how different super-voxel dimensions affect the shape of the resulting pliODF representations. Therefore, super-voxel dimensions close to the (fiber) structure dimensions (here: about 20 pixels, equivalent to 36 microns; cf. Figure 3A) were of specific interest. The binning of the directional histogram was set to (9 latitudes × 18 longitudes + 2 polar caps) = 164 bins.

As demonstrated in Figures 3A–C on the basis of an exemplary 40 × 40 × 1 super-voxel, the implemented resampling procedure of high-resolution FOMs provided a comprehensible description of the distribution of fiber orientations, in form of a directional histogram or a pliODF. The comparison of the pliODFs based on different levels of expansion with the corresponding directional histogram suggested that the approximation of the probability density function with spherical harmonics in the studied cases with the selected bin sizes should be confined (at least) to the 6th band, in order to reliably resolve orthogonal contributions (e.g., cyan and magenta).

**Figure 4:**
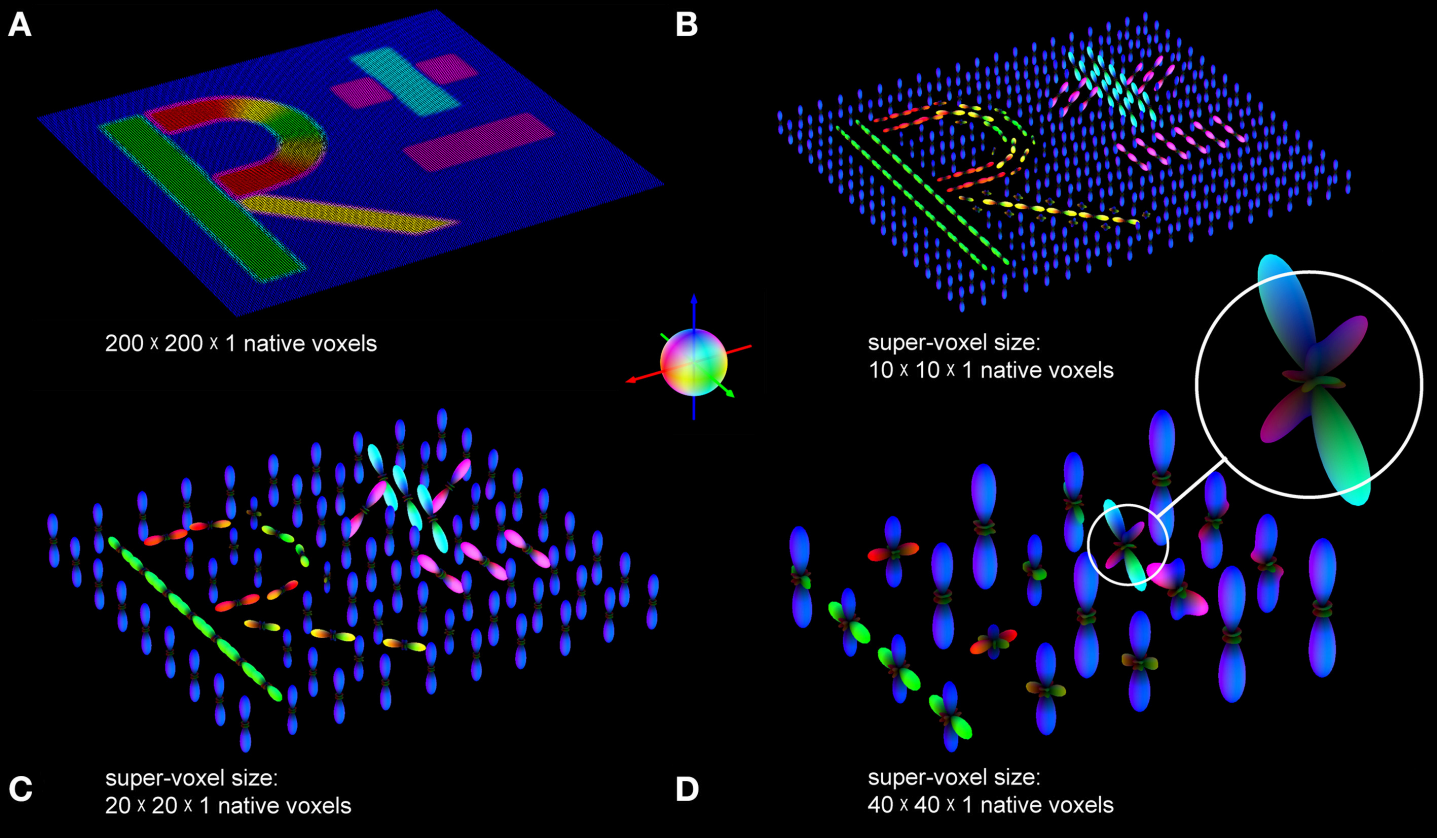
**pliODFs based on different super-voxel sizes. (A)** The FOM of the simulated dataset (cf. Figure 2) was divided into super-voxels composed of **(B)** 10 × 10 × 1, **(C)** 20 × 20 × 1, and **(D)** 40 × 40 × 1 native voxels. pliODFs were generated by means of series expansions to the 6th band for the different super-voxels. The color sphere defines the relation between orientation and color-coding.

The resampling results for different super-voxel dimensions are shown in Figures 4B–D. Compared to the original unit vector description of the fiber orientations, the peaks of the pliODFs obtained from the small super-voxels (Figures 4B,C) reflect the main underlying (microscopic) fiber orientations corroborated by the matching colors. In addition, the general (macroscopic) orientations of the letters agree with the orientations of the input structures. As expected, the complexity of the pliODF shapes increases in larger samples (Figure 4D), maintaining the major portions of fiber orientations.

### 3D-PLI data obtained from a hooded seal and a human brain

#### Brain sections of a hooded seal

**Figure 5:**
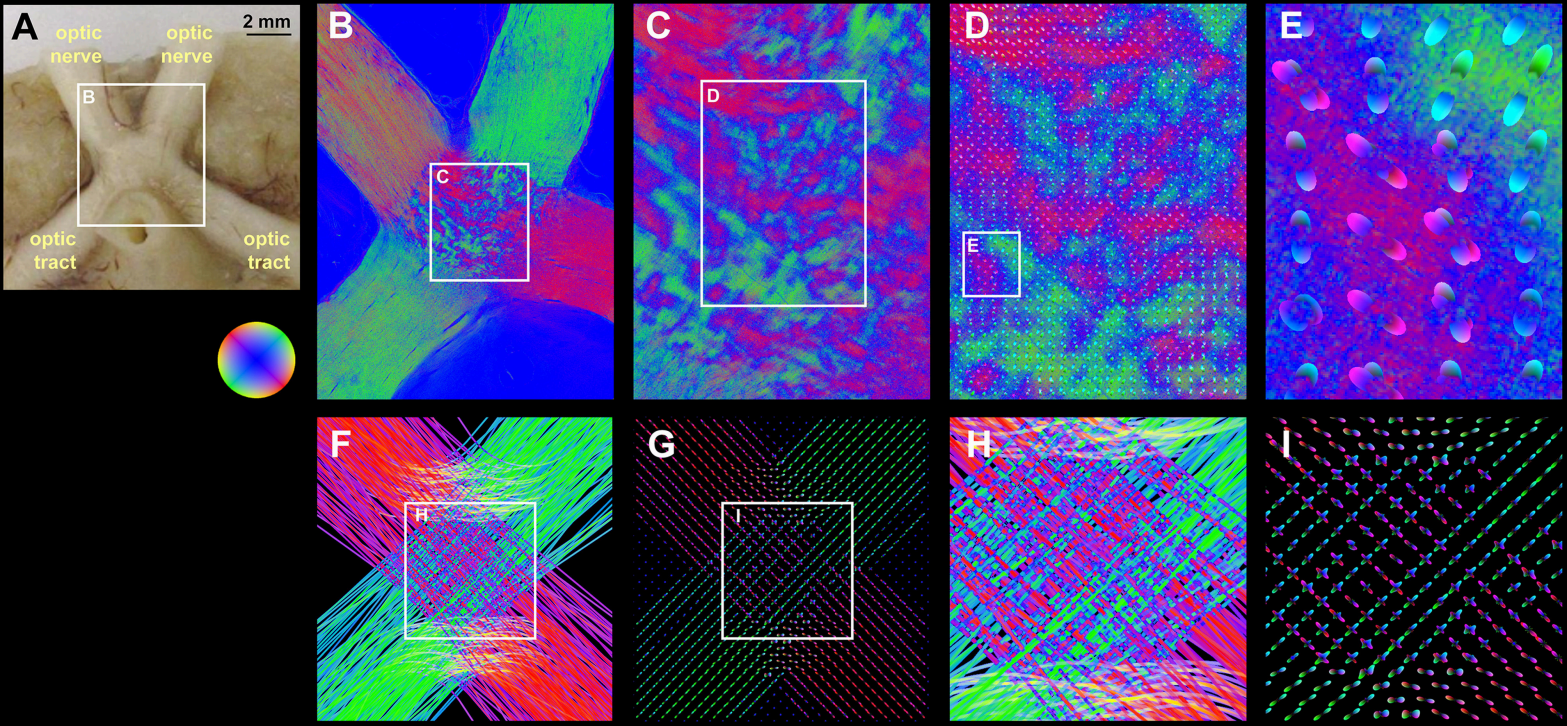
**Real and simulated brain section from the hooded seal. (A)** Blockface image of the optic chiasm of the hooded seal before sectioning. **(B)** Fiber orientation map of a medial section through the optic chiasm. Optic nerves and optic tracts appear as massive and rather homogeneous fiber bundles. Most fiber tracts from the optic nerves decussate to the contralateral optic tract. **(C)** The decussation zone in the center (i.e., the chiasm) is characterized by a patch pattern produced by small fiber tracts (red and green color; exemplary orientations are indicated by black lines) and fiber crossings characterized by signal attenuation (blue color; exemplary highlighted by white arrow). Based on this FOM, pliODFs were created for super-voxel dimensions of 40 × 40 × 1 native voxels. **(D,E)** demonstrate different enlargements of the field of pliODFs overlaid with the input FOM. **(F)** FOM of a simulated section through the optic chiasm and **(G)** corresponding pliODFs for super-voxel dimensions of 40 × 40 × 1 native voxels. **(H)** Zoom into the FOM of the fiber decussation zone and **(I)** corresponding pliODFs. The effects of crossing and bending fibers on the ODF shapes are obvious.

FOMs taken from hooded seal brain tissue show the optic nerve traversing through the optic chiasm into the optic tract (Figures 5A,B). The center of the optic chiasm reveals decussate fiber populations alternating with blue dots caused by signal attenuation (white arrow, Figure 5C). A pattern of crossing fiber tracts was used to prove the functionality of our implementation in a realistic setting. The FOMs were sampled using a histogram binning of (50 latitudes × 100 longitudes + 2 polar caps) = 5002 bins with a super-voxel size of 52 × 52 × 70 μm^3^, which is equivalent to 40 × 40 × 1 native voxels. The series expansion of the pliODFs was confined to the 6^th^ band. The fused images of the high-resolution FOMs with the corresponding pliODFs demonstrated a sound resampling (Figures 5D,E). The simulated chiasm of the hooded seal was analyzed accordingly and showed concordant results (Figures 5H,I).

#### Human brain sections

**Figure 6:**
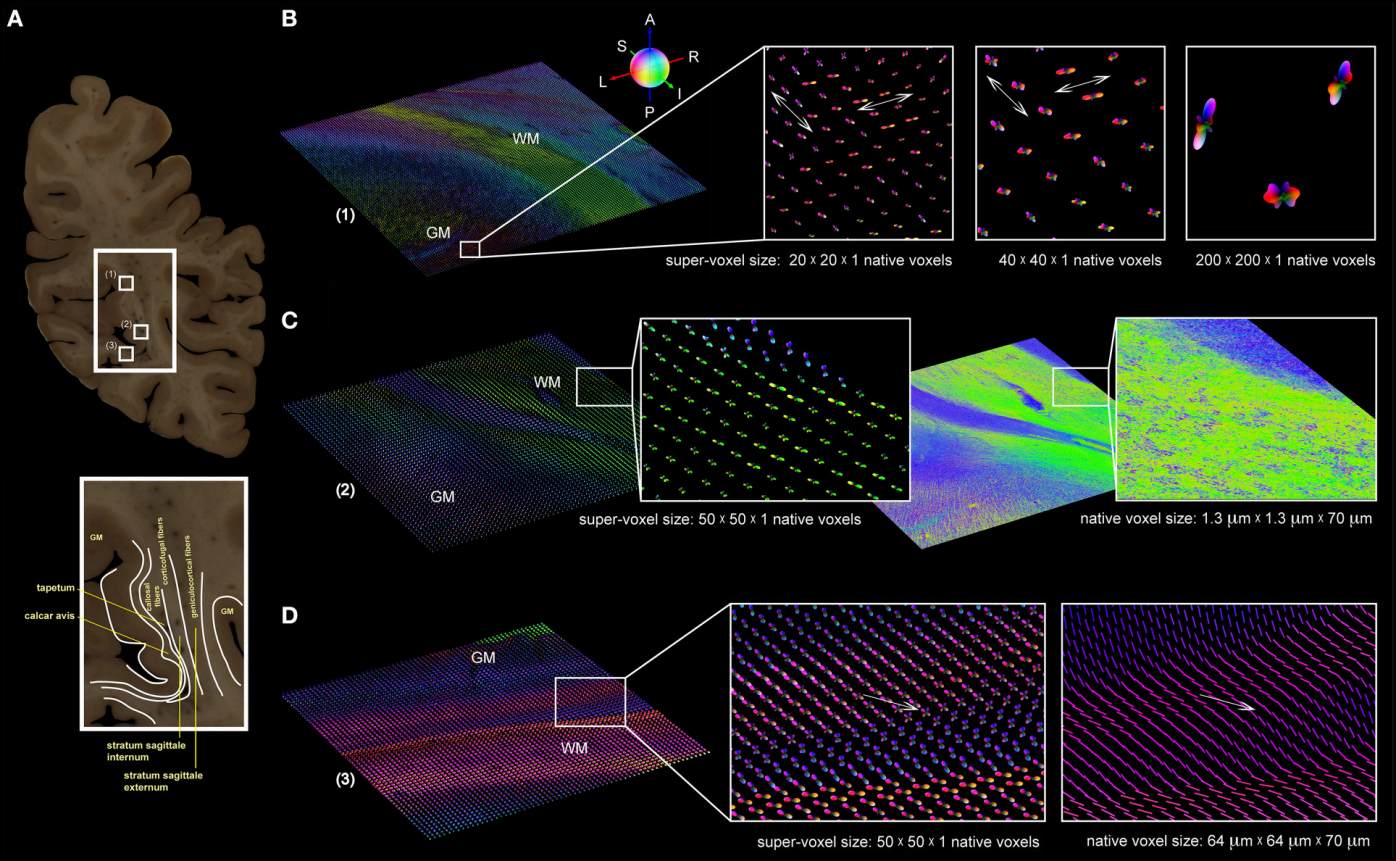
**Brain section from the human occipital lobe. (A)** Segmented blockface image acquired from the surface of the frozen human occipital lobe during the sectioning process. The small white rectangles (1) to (3) indicate the selected regions of interest for which pliODFs were determined (cf. **B–D**). The enlarged extract shows the delineation of anatomical structures, such as the tapetum, the calcar avis, and the stratum sagittale. **(B)** pliODF representations in region (1) with super-voxel dimensions of 20 × 20 × 1, 40 × 40 × 1 and 200 × 200 × 1 native voxels; the magnified images show the same cortical region, which is characterized by crossing fibers (indicated by the white arrows). The largest super-voxel size is equivalent to 260 × 260 × 70 μm^3^ and corresponds approximately to the level of high-resolution post mortem dMRI measurements. **(C)** Region (2) demonstrates for a super-voxel dimension of 50 × 50 × 1 native voxels the preservation of the overall fiber structure in comparison with the original high-resolution FOM obtained with the polarizing microscope. Zooming into the data reveals pliODFs with multiple fiber orientations in inhomogeneous white matter regions. **(D)** For region (3), pliODFs (super-voxel dimension of 50 × 50 × 1 native voxels) are opposed to the vector-based representation of the FOM of the same brain region measured with the large-area polarimeter at 64 × 64 × 70 μm^3^ voxel size. The white arrows indicate a crossing zone of fibers.

Three high-resolution FOMs of selected regions of interest from a coronal section through the human occipital lobe (Figure 6A) were resampled at different super-voxel dimensions (Figure 6B), but with fixed histogram binning (50 latitudes × 100 longitudes + 2 polar caps = 5002 bins). The targeted super-voxel sizes of 26 × 26 × 70 μm^3^, 52 × 52 × 70 μm^3^, and 260 × 260 × 70 μm^3^ correspond to 20 × 20 × 1, 40 × 40 × 1, and 200 × 200 × 1 native voxels, respectively. The series expansion was confined to the 6th band. The pliODFs were compared both with the underlying high-resolution FOMs acquired with the polarizing microscope (Figure 6C) and FOMs obtained with the large-area polarimeter (Figure 6D).

**Figure 7:**
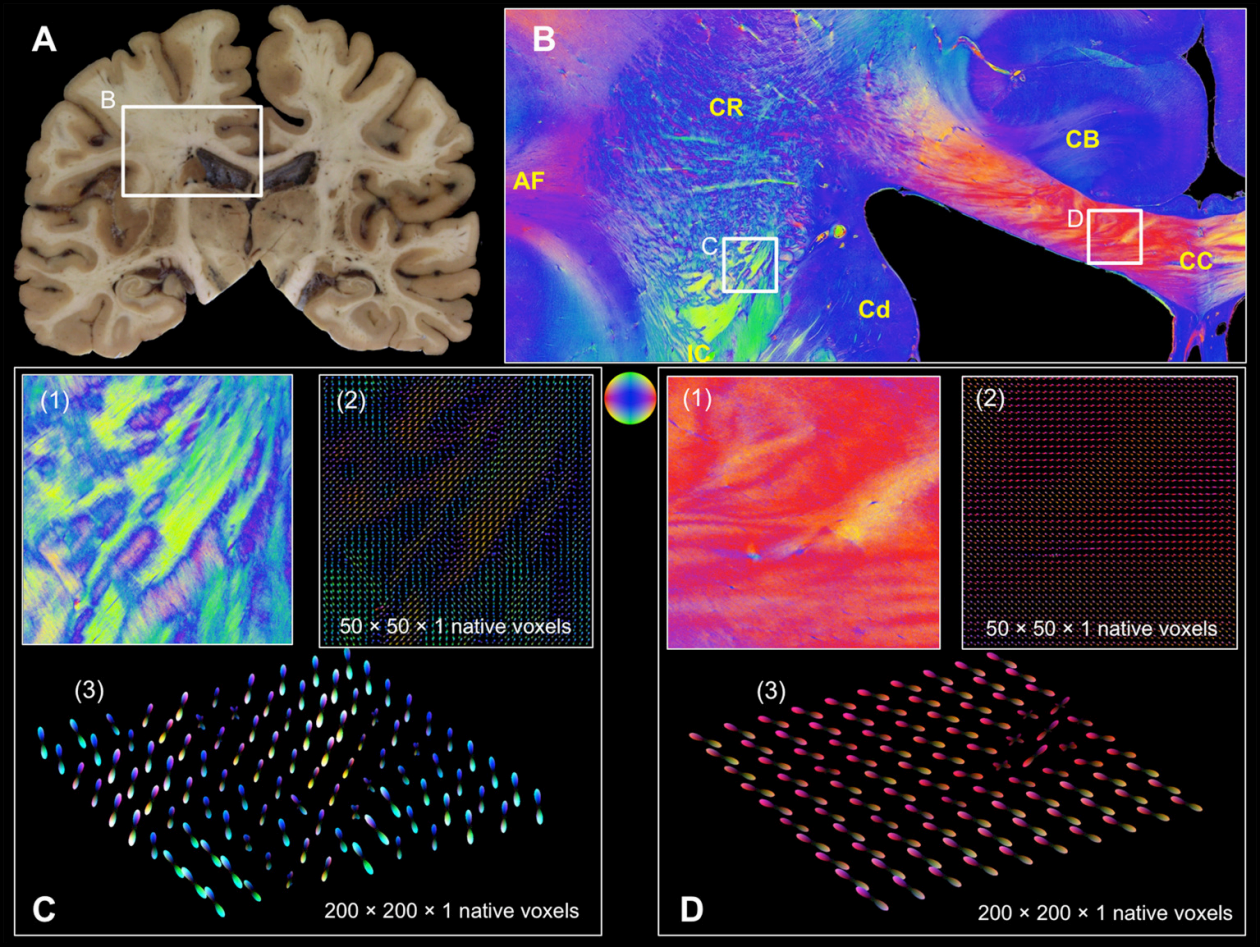
**Coronal section from the central human brain region. (A)** Segmented blockface image acquired from the surface of the frozen human brain. **(B)** Fiber orientation map comprising arcuate fascicle (AF), cingulum bundle (CB), corpus callosum (CC), caudate nucleus (Cd), corona radiata (CR), internal capsule (IC). Fiber orientations are RGB color-coded (see sphere). The white rectangles indicate FOMs (C(1) and D(1)) that were transferred into pliODFs. **(C)** pliODF representations in the region of CR/IC with super-voxel dimensions of (2) 50 × 50 × 1 and (3) 200 × 200 × 1 native voxels (from different views). Patches of crossing fiber bundles are clearly visible in the FOM and the pliODF maps. **(D)** pliODF representations in the region of CC with super-voxel dimensions of (2) 50 × 50 × 1 and (3) 200 × 200 × 1 native voxels (from different views). Although a predominant fiber direction is observable, small wriggling fiber bundles cause local inhomogeneities along the CC.

In addition, two high-resolution FOMs of selected regions of interest from a coronal section through the human brain (Figure 7) at the level of the central region were resampled at different super-voxel dimensions (Figures 7C,D), but with fixed histogram binning (50 latitudes × 100 longitudes + 2 polar caps = 5002 bins). The targeted super-voxel sizes of 65 × 65 × 70 μm^3^, and 260 × 260 × 70 μm^3^ correspond to 50 × 50 × 1, and 200 × 200 × 1 native voxels, respectively. The series expansion was confined to the 6th band.

The following observations were made:

- The overall fiber architecture in all regions of interest were preserved (Figures 6B–D, 7C,D) by pliODFs. This holds true for deep white matter tracts (e.g., in the stratum sagittale, Figure 6D, or the corpus callosum, Figure 7D). In cortical regions, the integrity of the fiber architecture is maintained by the 6^th^ band expansion even at increasing super-voxel sizes beyond 250 μm (Figure 6B).
- The peaks of the pliODFs, i.e., the prevailing directions, agree with the FOMs gained from the large-area polarimeter in tissue regions basically composed of parallel fibers (Figure 6D).
- Super-voxels localized in transition areas of adjacent or crossing fiber tracts preserved the corresponding fiber orientations in the pliODF with high fidelity (Figures 6D, 7C), while the same regions scanned with the large-area polarimeter showed attenuated signals or averaged orientations (e.g., Figure 6D).

#### Runtime behavior

**Figure 8:**
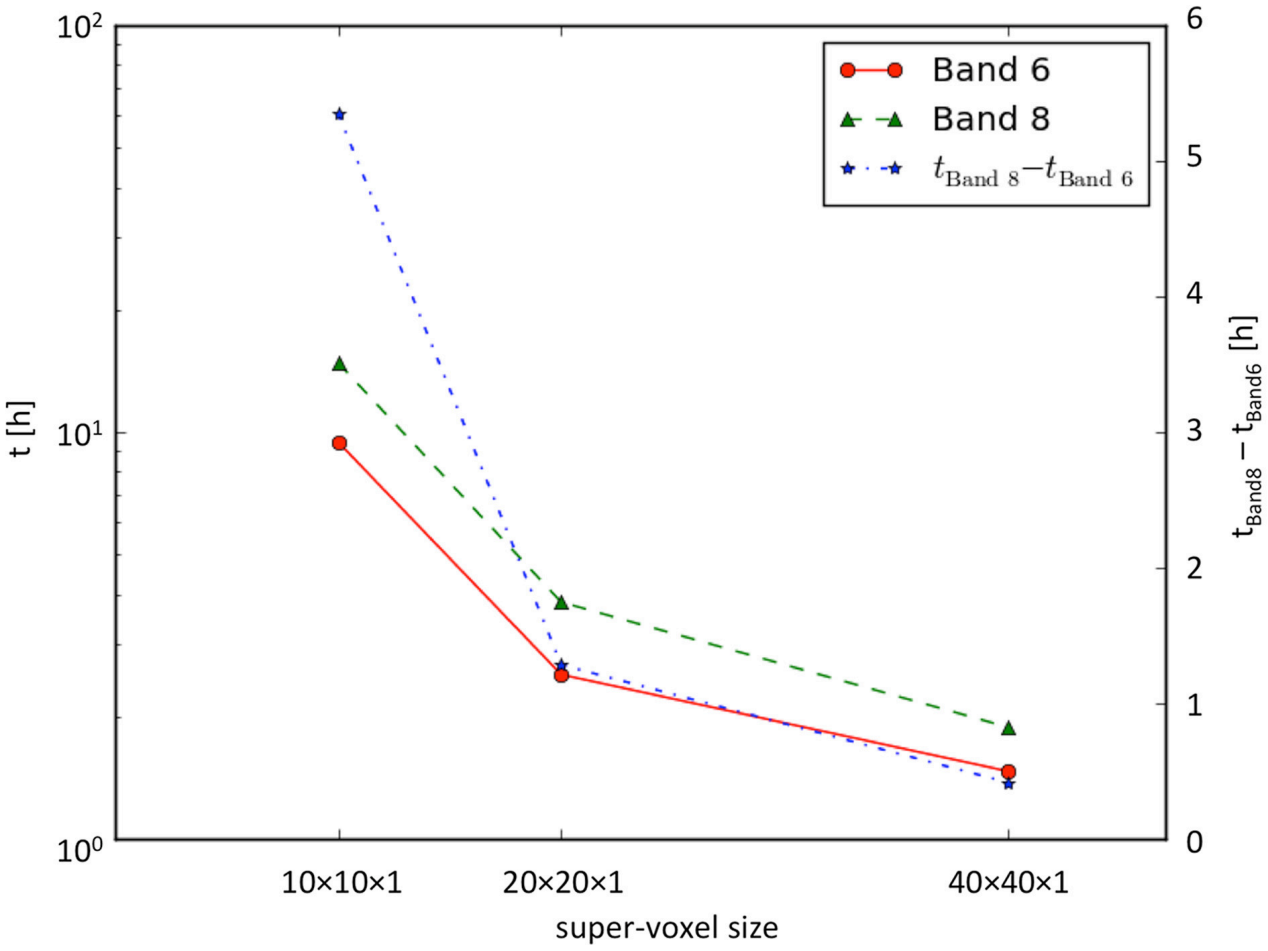
**Runtime measurements**. Runtime as a function of the super-voxel dimension for the series expansion up to the 6th and the 8th band. Note, that the left time axis is scaled logarithmically, while the right time axis is scaled linearly.

Runtime measurements (for 3712 × 4576 orientation vectors) performed on the JuDGE supercomputer showed that (i) with increasing super-voxel size the overall runtime decreased and (ii) with increasing depth of expansion the overall runtime increased (cf. Figure 8).

## Discussion

### General concept

3D-Polarized Light Imaging has been demonstrated in previous neuroanatomical studies (Axer et al., 2011a,b; Caspers et al., 2015; Dohmen et al., 2015; Zilles et al., 2015; Zeineh et al., 2016) to provide unique high-resolution data on the brains' fiber architecture of various species, such as mouse, rat, seal, vervet monkey, and human. The color-coded fiber orientation map (FOM) was the fundamental image modality for each of these studies, since it comprised both the highlighted fiber structures and their local 3D orientations. Hence, each FOM enabled a comprehensive delineation and identification of neuroanatomical fiber structures across different scales covering the micrometer (i.e., cortical and sub-cortical fibers) to the centimeter range (i.e., long-range fiber tracts across entire brain sections).

In the present study, we have successfully implemented a dedicated methodology of integrating high-resolution vector data obtained from FOMs into a comprehensive statistical description, the orientation distribution functions or pliODFs, respectively. By this means, efficient downscaling of high-resolution FOMs was achieved and cross-scale, cross-modality comparisons were enabled. The general concept of pliODF generation can be summarized in three steps:

- the definition of super-voxels (rectangular compartments) in high-resolution FOMs to resample the dataset,
- the collection of all fiber orientation vectors comprised in a super-voxel to derive the discretized distribution of orientations by creation of a normalized directional histogram on a unit sphere, and
- the approximation of the orientation probability distribution density by fitting the directional histogram with a series expansion using spherical harmonics. This yields a 3D glyph per super-voxel.

### Validation strategy

To prove the concept, we applied our implementation to simulated and real 3D-PLI datasets that reflected different characteristics of fiber compositions: well-defined parallel fibers (letter simulation, Figure 4), perpendicular fiber tract crossings (measurement and simulation of the hooded seal chiasm, Figure 5), and complex fiber architectures (measurements in human brain sections, Figures 6, 7). That way, the feasibility of our approach became traceable from the most simple to the most challenging cases.

The reliability of the steps required to derive a pliODF in form of a glyph was successfully demonstrated on basis of a simulated 3D-PLI dataset. The simulation tool SimPLI (Dohmen et al., 2015; Menzel et al., 2015) has specifically been developed to model the effects of birefringence in brain tissue as well as the results of polarimetric measurements. However, one of the generated synthetic datasets (cf. Figure 2) was not destined for realistic simulation of fiber architecture, but to enable systematic testing of the conservation of the involved coordinate systems, and fiber-like/bundle-like orientations, respectively. In combination with the same color-coding scheme applied to FOMs and pliODFs, this turned out to be an excellent approach for demonstration and validation purposes. This becomes evident in the study of directional histograms (i.e., the probability distributions of orientation vectors on a sphere) and their fitting with a series expansion with spherical harmonics (Figure 3).

The directional histogram is a proximate reflection of the main fiber orientations in a super-voxel as provided by the FOMs. However, the discrete arbitrary binning of the histogram affects the orientation information and, consequently, the final approximation of the density function. The different bin widths for simulation and experimental datasets, which were used for this study resulted from trials. For future studies, an automated data driven binning in S^2^, depending on input and target data, is essential. The creation of directional histograms from high-statistics vector data is not computationally intensive and represents a very fast and robust method to derive the prevalent fiber directions in super-voxels. The precision of the fiber directions obtained from the directional histogram (defined by the bin centers) is basically limited to the size of the dihedral angle spanning a bin.

This limitation can be overcome by fitting the directional histogram with a series expansion using spherical harmonics to generate a pliODF, but at the expense of computing time that is increasing drastically. As derived from runtime measurements, the resampling of a FOM composed of 3712 × 4576 orientation vectors, the computationally most intensive step was the determination of the expansion coefficients. According to Equation 7, the number of coefficients to be determined increases non-linearly with increasing expansion depth. Hence, the series expansion has to be truncated. For the experiments done in this study, the expansion to the 6^th^ band appeared to be sufficient to assess the functionality of our implementations, but, this level of resolution has to be reviewed in case of addressing different questions as to data precision, data size or computing time. By increasing the super-voxel size, the computation time is decreased in a non-linear way, due to the lower number of pliODFs to be computed.

What does this mean for whole human brain imaging and comparison? A human brain volume of 1200 cm^3^ translates into 10^10^ native voxels provided by high-resolution 3D-PLI measurements. A voxel size of 2 mm isotropic, such as provided by standard clinical *in vivo* dMRI technologies (e.g., Bastiani and Roebroeck, 2015), leads to 150,000 dMRI voxels for a human brain. Resampling of 3D-PLI to this dMRI scale means integrating 65,000 orientation vectors into a single super-voxel or pliODF, respectively. For the runtime measurements performed on the 3712 × 4576 pixel-sized FOM, about 170,000 pliODFs with a super-voxel dimension of 10 × 10 × 1 native voxels had to be computed. The computation time (expansion to the 6th band) was about 9.5 h utilizing only 72 compute cores. Conclusively, computation of pliODFs for an entire human brain at 2 mm resolution is feasible. Post mortem dMRI has recently progressed to study the structural organization of the entire human brain at a voxel size of 0.7 mm isotropic (Miller et al., 2011), which results in 3.5 million target (super-)voxels, which is a factor of 20 more than provided by standard *in vivo* dMRI measurements. However, adjusting pliODFs to submillimeter dMRI data for a whole human brain is still in the realm of the feasible, taking into account that supercomputing facilities provide thousands of computing cores.

### Scope of application

We demonstrated that the pliODF generation workflow enables the study of scaling effects and related partial volume effects efficiently, already at the level of single brain sections for both real and simulated data (cf. Figures 5–7). 3D-PLI measurements of the same tissue at two distinct resolutions (native voxel sizes of 64 × 64 × 70 μm and 1.3 × 1.3 × 70 μm) were beneficial in this context. The low-resolution FOMs were used as independent references for prevailing fiber orientations derived from pliODFs computed from super-voxel dimensions that matched the native voxel dimensions of the low-resolution measurements (e.g., Figure 6D). While the principal orientations of distinct fiber tracts from both types of fiber orientation descriptions agreed, the benefit of pliODFs became evident at transition zones between differently oriented fiber tracts. In the latter case, pliODFs preserved details about the complex fiber population, which was not observed for the low-resolution measurements. This is due to the fact, that in a 3D-PLI measurement a native voxel collects birefringence effects from multiple fibers resulting in a measurement of superimposed sinusoidal signals (Reckfort et al., 2015), each with fiber orientation specific amplitude and phase. As demonstrated by Dohmen et al. (2015), the derived fiber orientation vector for a native voxel significantly depends on the complexity of the underlying fiber population. For the low-resolution measurements this means that about 50–100 myelinated fiber contribute to the measured signal, if fiber diameters between 0.4 and 15 μm (Aboitiz et al., 1992) are assumed. Future studies will further elaborate on scaling effects by combining sophisticated simulation approaches (Dohmen et al., 2015; Menzel et al., 2016) with measurements across scales (Reckfort et al., 2015). This will open up new avenues to derive observer independent quality measures for 3D-PLI measurements, beyond neuroanatomical inspection.

Even though the concept of the pliODF generation has been demonstrated for FOMs of individual brain sections, its application is not limited to section-like data, but it can also be extended to a volume of FOMs. In the latter case, the brain section images have to be re-aligned into a coherent 3D brain volume, which requires application of complex non-linear image registration techniques (Palm et al., 2010; Amunts et al., 2013). Assuming a whole human brain reconstruction from 2500 serial coronal sections scanned at 1.3 μm pixel size with a single section image size of 70,000 × 100,000 pixels on average, it becomes evident, that the utilization of distributed high performance computing on a supercomputing environment is essential. The process of registration aims at correcting for up to cm-sized tissue deformation introduced during brain sectioning and tissue mounting. This poses a major challenge when addressing 3D brain reconstruction at the μm-scale, as required for long-range pixel-wise tracing of fiber tracts across hundreds to thousands of brain sections. By integrating many orientation vectors into pliODFs, local inaccuracies in section alignment are likely to be polished and, therefore, tractography of long distance fiber pathways become feasible for large-scale 3D-PLI datasets, but at the expense of resolution. As a benefit, advanced tractography algorithms exploiting multiple direction information of individual dMRI voxels (Sotiropoulos et al., 2010; Reisert et al., 2011) become applicable to 3D-PLI data and, vice versa, pliODFs (or, alternatively, directional histograms) are suitable to validate various methods of tractography.

As pointed out by Hubbard and Parker (2009), “it is important to …test not only the ability of the tractography algorithms to track fibers from voxel to voxel, but to observe the details of the voxel-scale information and independently quantify the ability of dMRI to assess fiber orientation. The integrity of this orientation information is paramount to the validity of the reconstructed tract.” 3D-PLI with its pliODFs particularly opens up the avenue to align with dMRI measurements by crossing the scales using common data formats, and to provide sub-voxel information on the underlying fiber architecture based on an independent technology complementary to the dMRI approach. Based on the pliODF generation, comparisons of 3D-PLI and dMRI can now be conducted at the level of individual voxels of the same size. Describing the local distribution of fiber orientations by means of spherical harmonics opens up the possibility to utilize methods being developed in the scope of computer vision to assess the similarity of datasets via shape descriptors and shape metrics, for example. This appears to be a promising approach to derive observer independent quality measures for 3D-PLI measurements, which will be evaluated in future studies.

## Conclusions

The future of research about the brain connectome will depend on multiscale approaches validated by independent imaging methods applied to the same object simultaneously. We have successfully established a concept to bridge the spatial scales from microscopic fiber orientation measurements based on 3D-PLI to macroscopic dimensions by means of creating orientation distribution functions (pliODFs) from high-resolution vector data. With pliODFs the fusion and comparison with dMRI data becomes feasible even for whole human brains. The key-technology of supercomputing, that is inevitable for addressing real big data challenges as posed by the human brain, has been applied to achieve this goal. Though, our implementation does not only limit the software's application to supercomputing environments, but it was also demonstrated to run successfully on local linux cluster systems. The established software package to generate pliODFs from 3D-PLI datasets described in this work will be made available through an ICT portal currently being developed by the human brain project consortium.

## Author contributions

MA coordinated and substantially contributed to the conception and design of the study as well as to the analysis and interpretation of the 3D-PLI data. He drafted the manuscript. SS participated in the design of the study and was accountable for the mathematical description of the pliODFs and the software implementation. He helped to draft the manuscript. DG contributed to the interpretation of the 3D-PLI data and was accountable for the visualization of the directional histograms and the pliODFs. He helped to draft the manuscript. OB implemented the HPC-based analysis workflow of 3D-PLI data and generated the pliODFs for the whole human brain section. MD created and analyzed the simulated data set. JR conducted the 3D-PLI measurements and helped to compare the high- and low-resolution data sets. KZ contributed to the anatomical content of the study and helped with the interpretation of the pliODFs. KA contributed to the anatomical content and substantially assisted to the conception of the study. All authors read and revised the final manuscript and gave approval for publication.

## Funding

This work was supported by the Helmholtz Association portfolio theme “Supercomputing and Modeling for the Human Brain,” by the European Union Seventh Framework Programme (FP7/2007-2013) under grant agreement no. 604102 (Human Brain Project), and by the National Institutes of Health under grant agreement no. R01MH0 92311.

### Conflict of interest statement

The authors declare that the research was conducted in the absence of any commercial or financial relationships that could be construed as a potential conflict of interest.
